# Supplementary figures and images for: Comprehensive mutational analysis of the checkpoint signaling function of Rpa1/Ssb1 in fission yeast
Source: PLoS Genet. 2023 May 18;19(5):e1010691. doi: 10.1371/journal.pgen.1010691 (PMC10231789; doi:10.1371/journal.pgen.1010691)

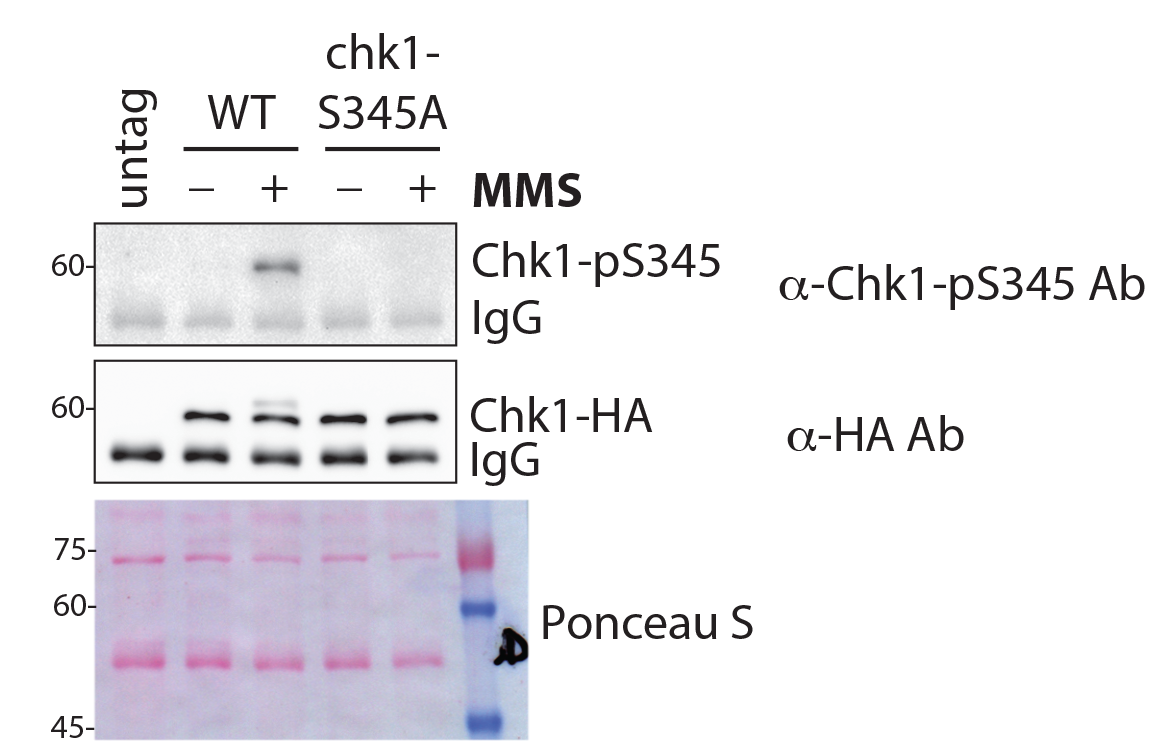

Supplement: S1 Fig — Logarithmically growing cells were treated with 0.01% MMS in YE6S medium for 90 min at 30°C. 5.0 OD cells were harvested from each culture and saved in a screw-cap microtube at -20°C. The frozen cell pellets were lysed by mini-bead beater in HEPES/NaOH buffer containing 150 mM NaCl and inhibitors of phosphatases and proteases. Chk1-HA was IPed using anti-HA antibody beads in cold room for 2 h. The IPed samples were separated on an 8% SDS-PAGE gel and then transferred to a nitrocellulose membrane. The membrane was strained with Ponceau-S to show the IgG bands between the 60 and 45 KDa markers (lower panel). The membrane was first incubated with the phospho-specific antibody at 1:3000 dilutions for 3 h at room temperature to reveal phosphorylated Chk1(top panel). After stripping, the membrane was extensively washed in deionized water and then reblotted with anti-HA antibody to reveal the HA-tagged Chk1 (middle panel). NW223 strain expressing HA tagged Chk1 was used as the wild-type cells, whereas NW444 expressing HA tagged Chk1-S345A was used as the mutant control. TK7 STRAIN expressing untagged Chk1 was used as the control for specific IP. (TIF) [file pgen.1010691.s001.tif]

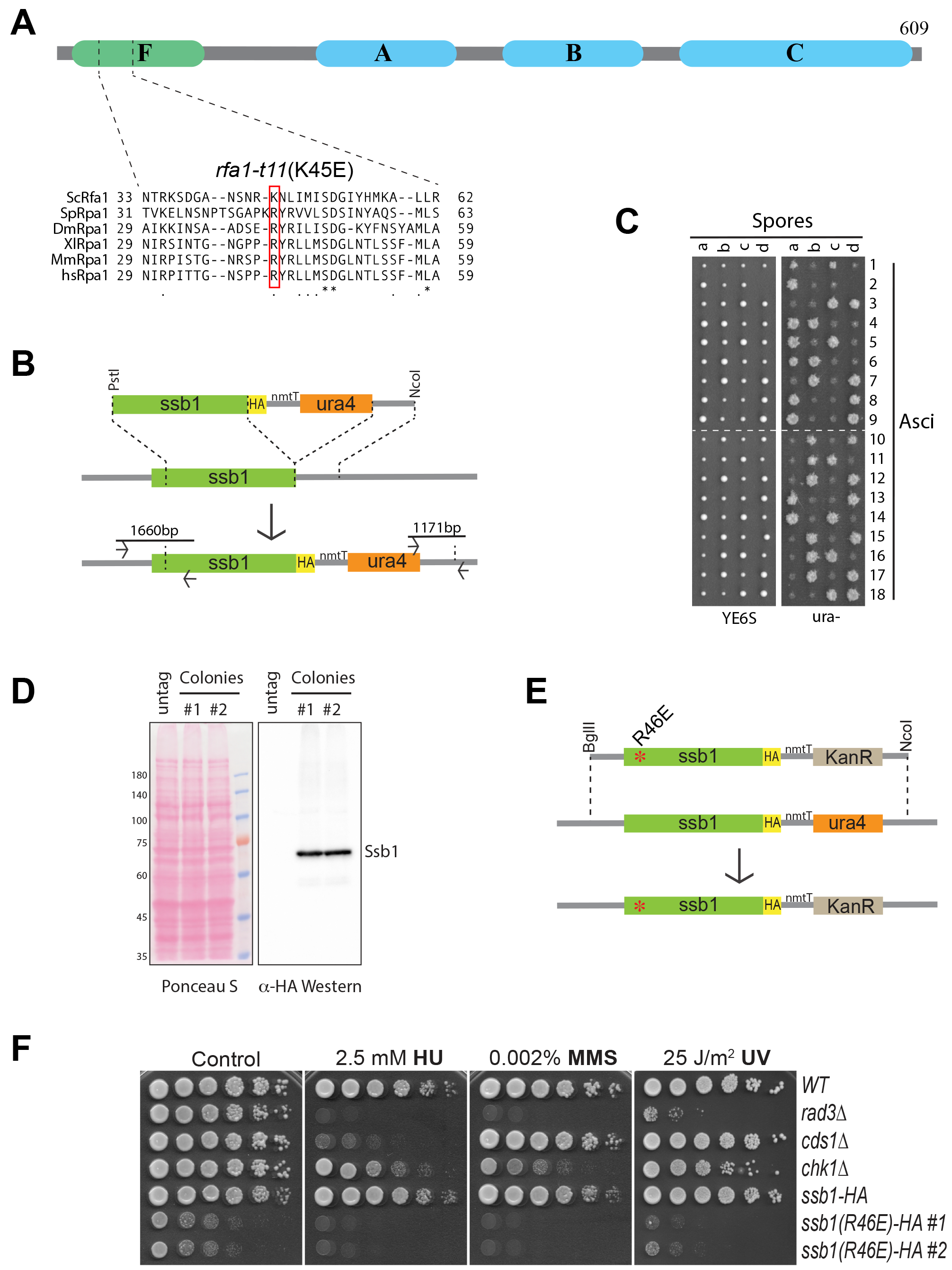

Supplement: S2 Fig — (A) Diagram of Ssb1, the F domain, and the rfa1-t11 mutation. The positively charged Lys45 residue mutated in rfa1-t11 is highly conserved from yeasts to humans. Sc: S cerevisiae, Sp: S. pombe, Dm: Drosophila melanogaster; Xl: Xenopus laevis, Mm: Mus musculus, and Hs: Homo sapiens. (B) Strategy for tagging ssb1 with an HA epitope linked with ura4 marker at the genomic locus. The resulting ura+ colonies were selected by colony PCR to confirm the correct 5’ and 3’ integrations. nmtT: nmt terminator. (C) The tagged strain was confirmed by tetrad dissection that showed 2:2 ratios of ura+ and ura- spores for all dissected asci. Dashed line indicates discontinuity. (D) The tagged strain was also confirmed by Western blotting of the whole cell lysates using anti-HA antibody. (E) The marker switching method for replacing wild type ssb1 at the genomic locus with the mutant ssb1-R46E. The ssb1-K45E mutation is likely lethal in S. pombe as its replacement did not generate any colonies (data not shown). (F) The ssb1-R46E mutant showed a severe growth defect on YE6S plate. (TIF) [file pgen.1010691.s002.tif]

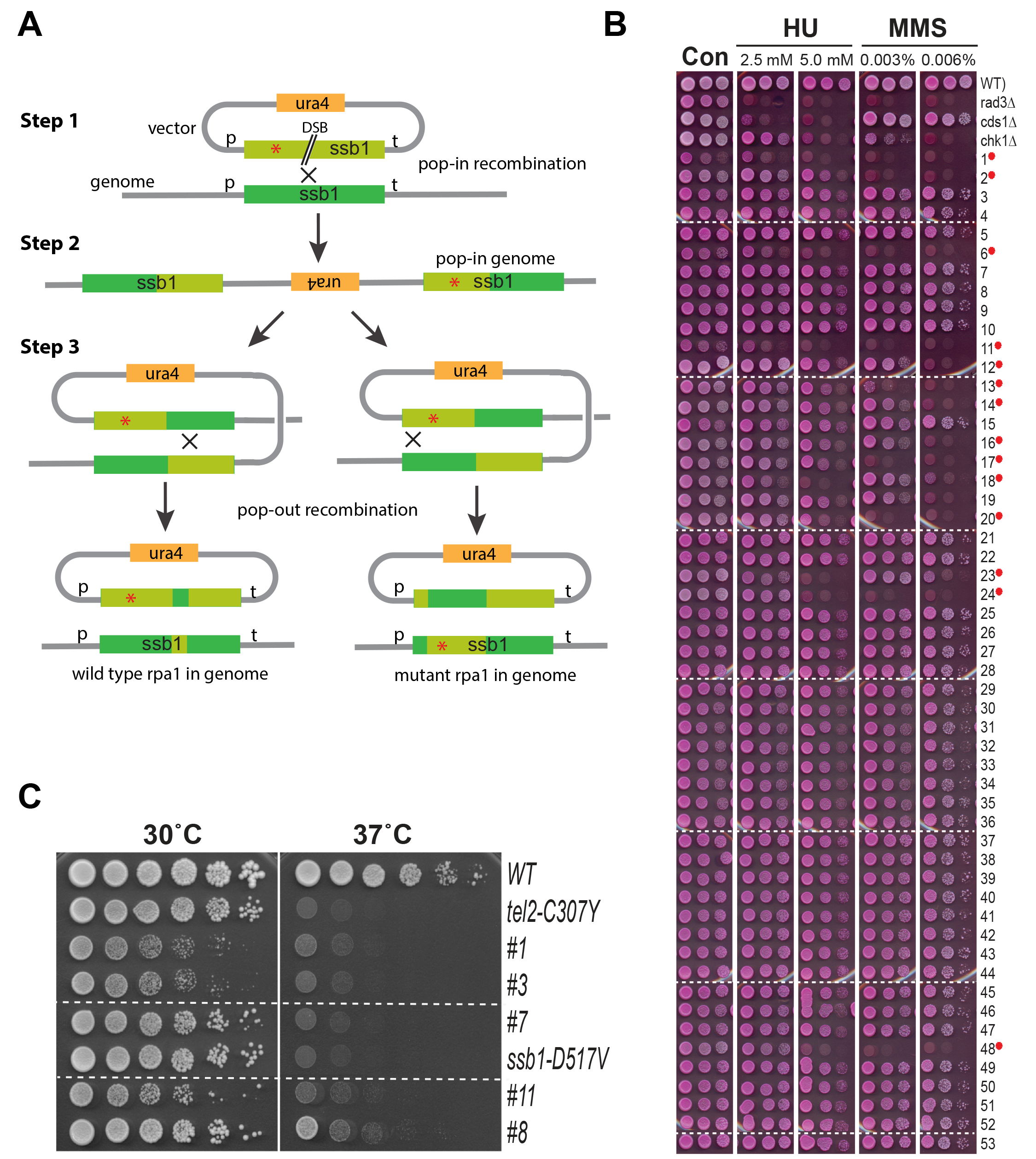

Supplement: S3 Fig — (A) The strategy for ssb1 replacement (55). The ORF of ssb1 was mutated by PCR at the N- and C-terminal halves separately to generate two libraries. After linearization by enzyme digestion, the library DNA is transformed into wild-type S. pombe lacking the ura4 gene. The cells were cultured in EMM6S[ura-] to select the transformants with the integrated ura4 marker. The ura4+ cells were then cultured in YE6S to pop-out the ura4 marker to be counter selected by 5-FOA. The colonies formed on 5-FOA plates carry either wild type or mutant ssb1 at the genomic locus. The ssb1 mutants were screened by replica plating on HU plates. (B) The screened mutants were streaked out into single colonies for confirming the drug sensitivities. As an example, the HU and MMS sensitivities of the primary mutants screened with N-terminal half library were assessed by three-spot assay. The drug sensitive mutants marked by red asterisks were backcrossed once, renamed, and then saved for further investigation. (C) The ts phenotype of some of the screened mutants was assessed by spot assay. The tel2-C307Y mutant, used as a control, is a ts mutant that we screened previously (39). (TIF) [file pgen.1010691.s003.tif]

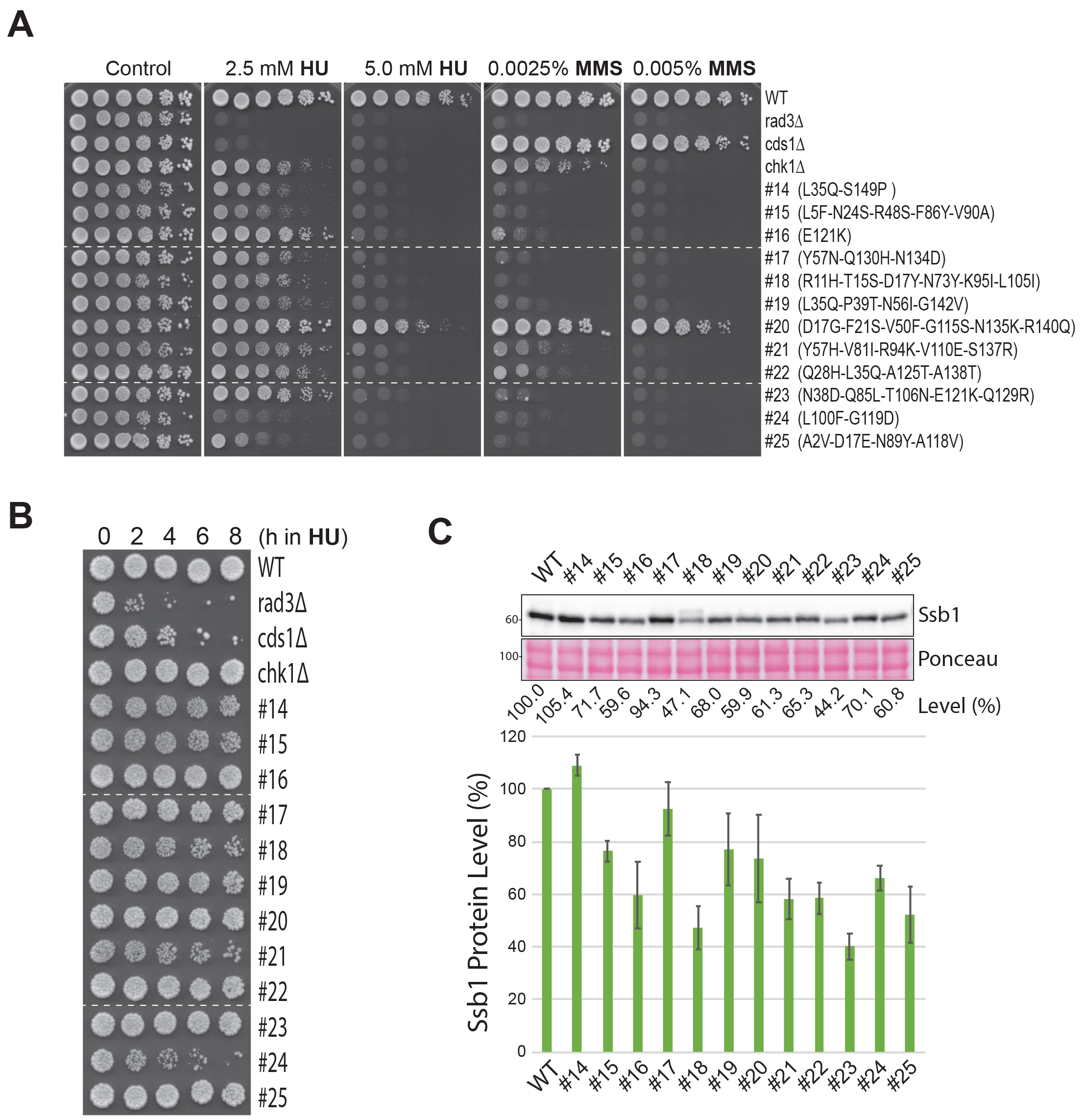

Supplement: S4 Fig — (A) Sensitivities of the twelve mutants (#14 - #25) to HU and MMS were examined by spot assay. The amino acid changes in the mutants are shown on the right. Dashed lines indicate discontinuity. (B) Acute HU sensitivity of the twelve ssb1 mutants was assessed by spot assay. Dashed lines indicate discontinuity. (C) Ssb1 protein levels were examined in logarithmically growing wild-type and the mutant cells by Western using anti-Ssb1 antibody (top panels). A section of Ponceau S-stained membrane is shown for loading. The Western blotting was repeated three times. Quantitation results are shown (lower panel). Error bars: means and SDs of the triplicates. (TIF) [file pgen.1010691.s004.tif]

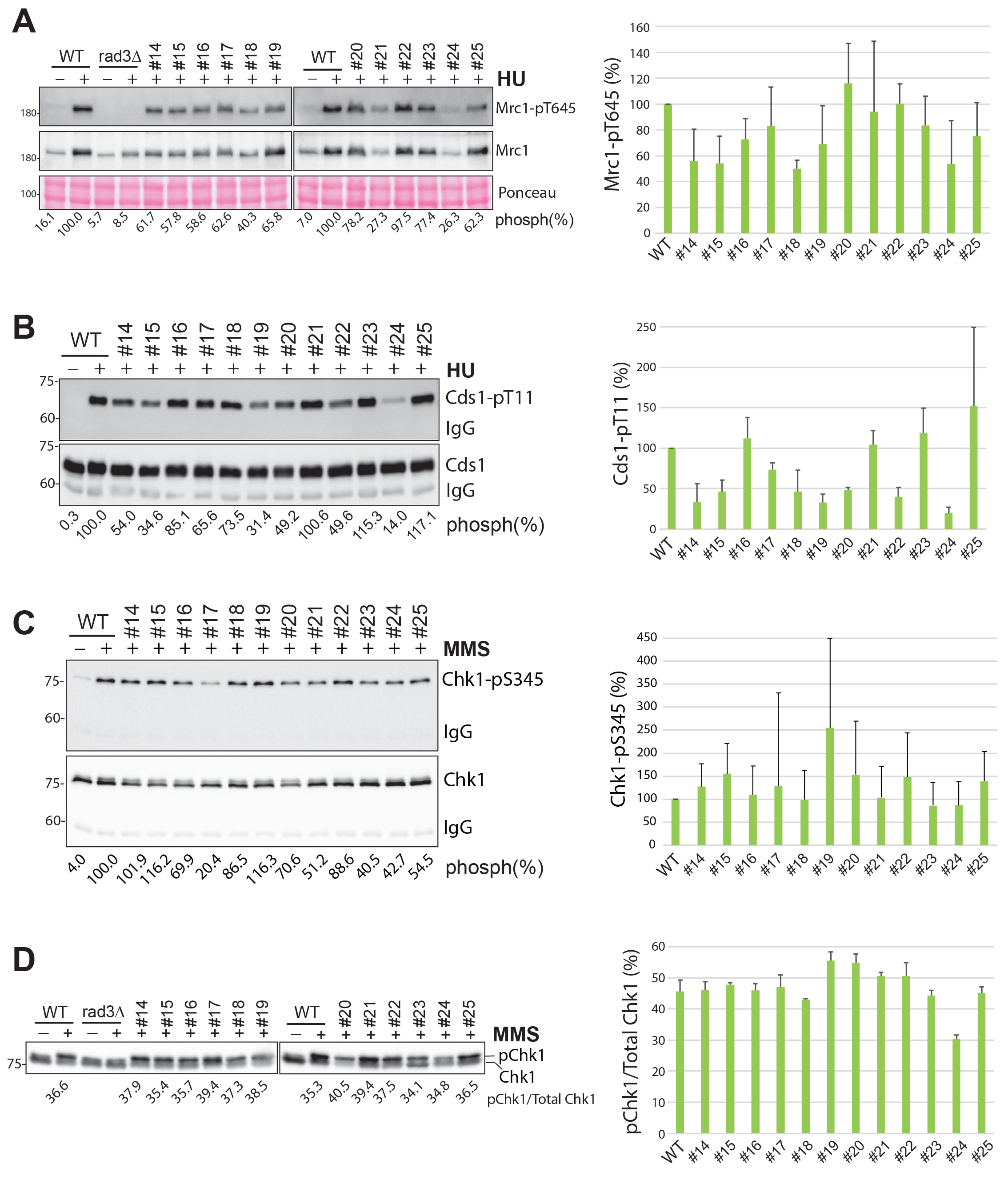

Supplement: S5 Fig — (A) Mrc1 phosphorylation by Rad3 was examined by Western blotting before (-) or after (+) the cells were treated with 15 mM HU for 3 h (left). Quantitation results are shown on the right. Error bars are the means and SDs of three independent blots. (B) Cds1 phosphorylation by Rad3 was examined by Western blotting in the mutants. Quantitation results are shown on the right. (C) Chk1 phosphorylation by Rad3 was examined by Western blotting using phospho-specific antibody against Chk1-pS345 after the cells were treated with 0.01% MMS for 90 min (left). Quantitation results are shown on the right. (C) Chk1 phosphorylation was also examined by the commonly used mobility shift assay. Wild-type and the mutant cells were treated with 0.01% MMS for 90 min. The cell lysates made by TCA method were analysed by an 8% SDS PAGE gel for Western blotting with anti-HA antibody. Quantitation results are shown on the right. (TIF) [file pgen.1010691.s005.tif]

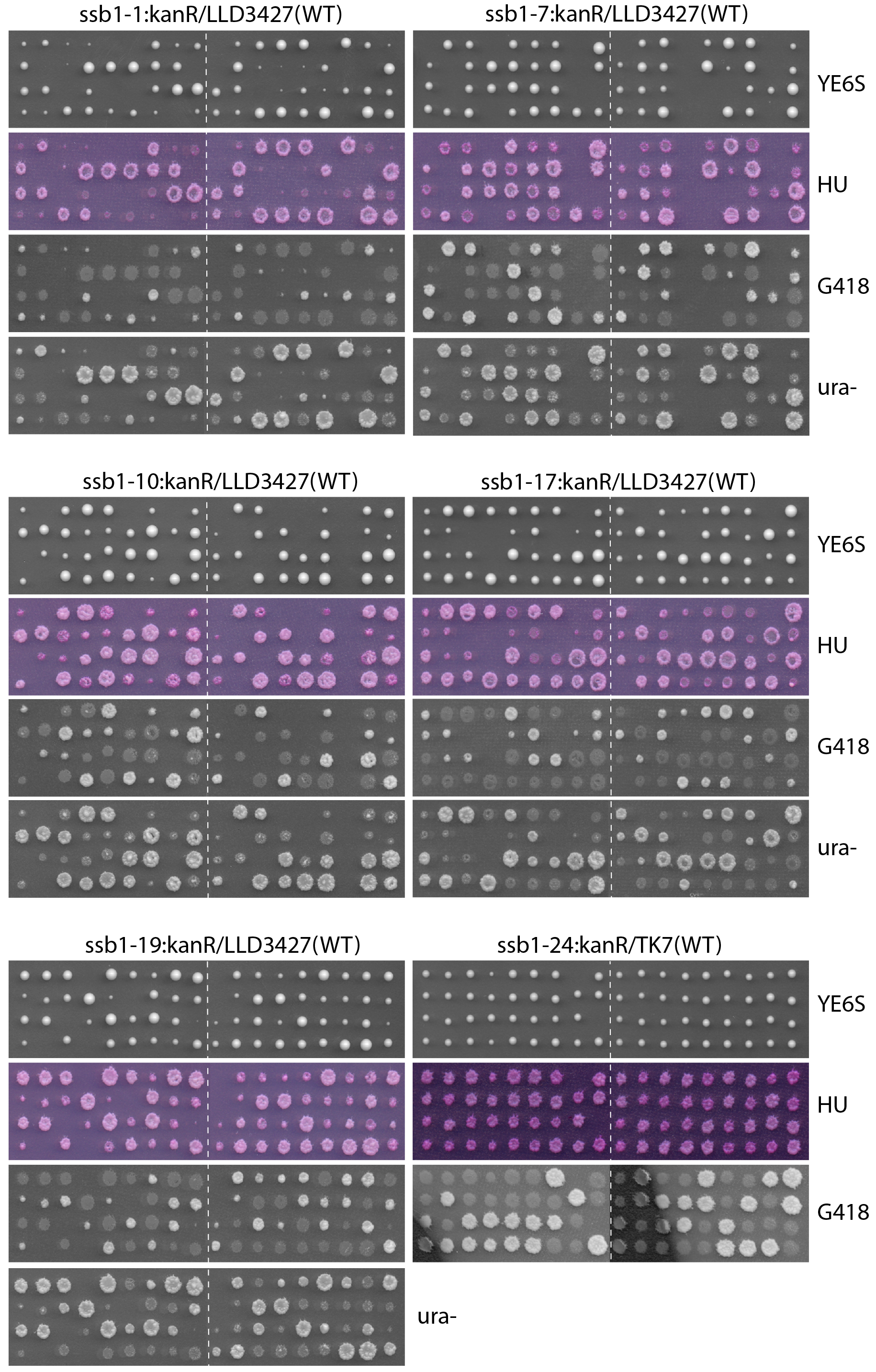

Supplement: S6 Fig — The untagged ssb1 integrants with ssb1-1, ssb1-7, ssb1-10, ssb1-17, ssb1-19, and ssb1-24 mutations linked to the kanR marker were made by using the marker switching method shown in S2E Fig. The integrants were backcrossed with the wild type LLD3427 strain carrying a ura4 marker or TK7 lacking the ura4 marker. Tetrad dissections were performed for each cross and colonies formed on YE6S plates were replicated onto plates containing 5 mM HU and the lethality dye phloxine B, YE6S plates containing 100μg/ml G418, and EMM6S[ura-] plates. All tetrads showed 2:2 ratios of kanR or ura+ spores and the hus phenotype is absolutely linked to the kanR marker in all integrants. Dashed lines: discontinuity. These results confirm single integration in the genome. (TIF) [file pgen.1010691.s006.tif]

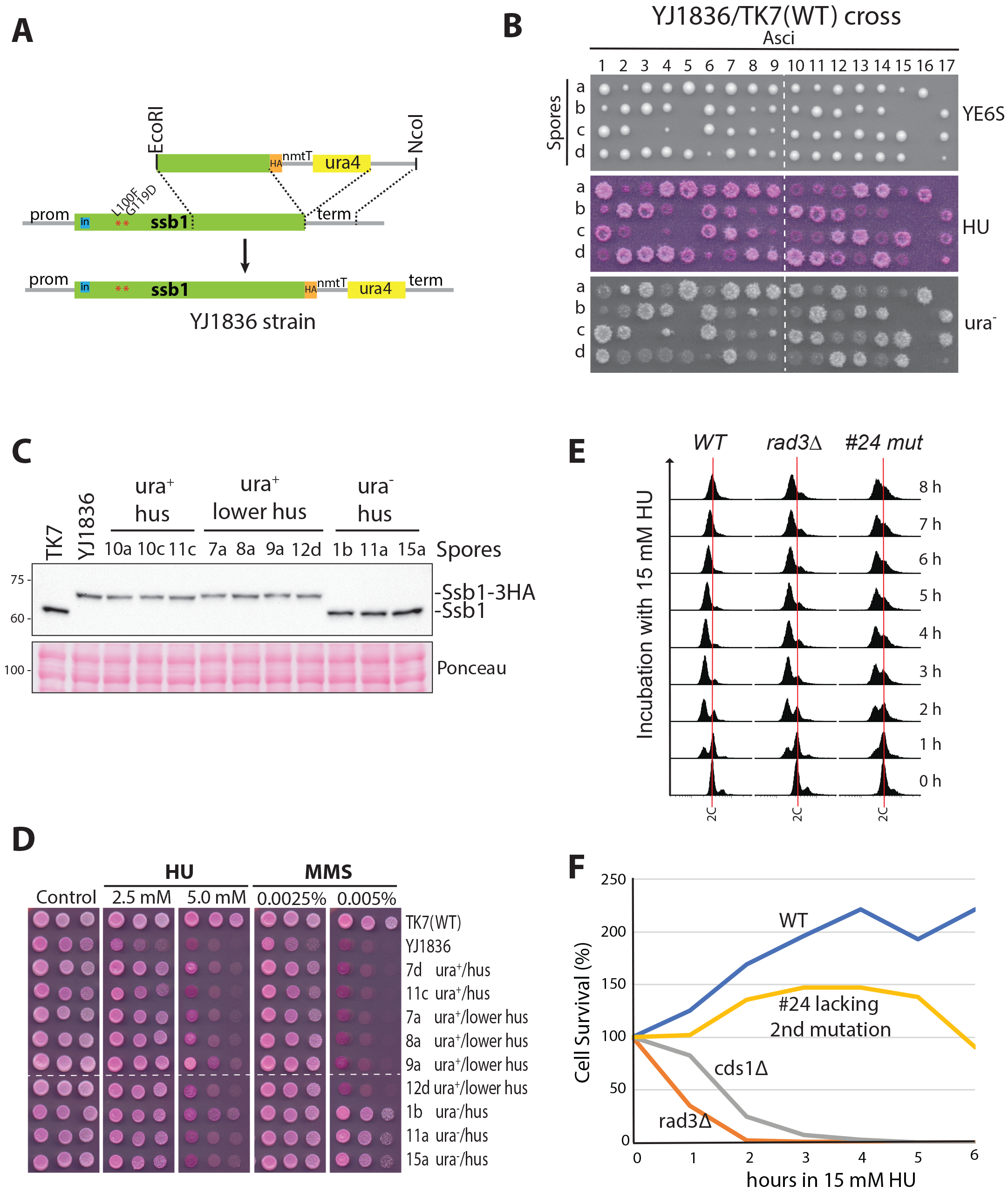

Supplement: S7 Fig — (A) Strategy for tagging the #24 mutant ssb1 with HA linked with a ura4 marker. nmtT: nmt terminator. (B) The tagged strain YJ1836 was crossed with the wild-type TK7 strain for tetrad dissection. Colonies were replicated onto HU and EMM6S[ura-] plates. This tetrad dissection identified three groups of spores with the hus phenotype. Spores in the first group are ura+ with severe hus phenotype such as the 10a and 11c spores. Those in the second group are ura+ with a lower HU sensitivity such as the 7a and 8a spores. Spores in the third group are ura- such as 1b and 11a. (C) Ssb1 levels in the representatives of the three groups were examined and compared with wild type TK7 and YJ1836 cells. (D) HU and MMS sensitivities of representative spores were assessed by spot assay. Note: the ura- spores in the third group are resistant to MMS, suggesting a secondary unknown metabolic mutation in the #24 mutant (50, 51). (D) Consistent with the metabolic mutation, HU arrested a large fraction of the #24 mutant cells in G2/M, not S phase, which explains the observed “checkpoint defect” in the DRC. (E) After removing the secondary mutation, the #24 mutant became insensitive to acute HU treatment as determined by colony recovery assay. Data points are means of the numbers of recovered colonies on three separate plates. (TIF) [file pgen.1010691.s007.tif]

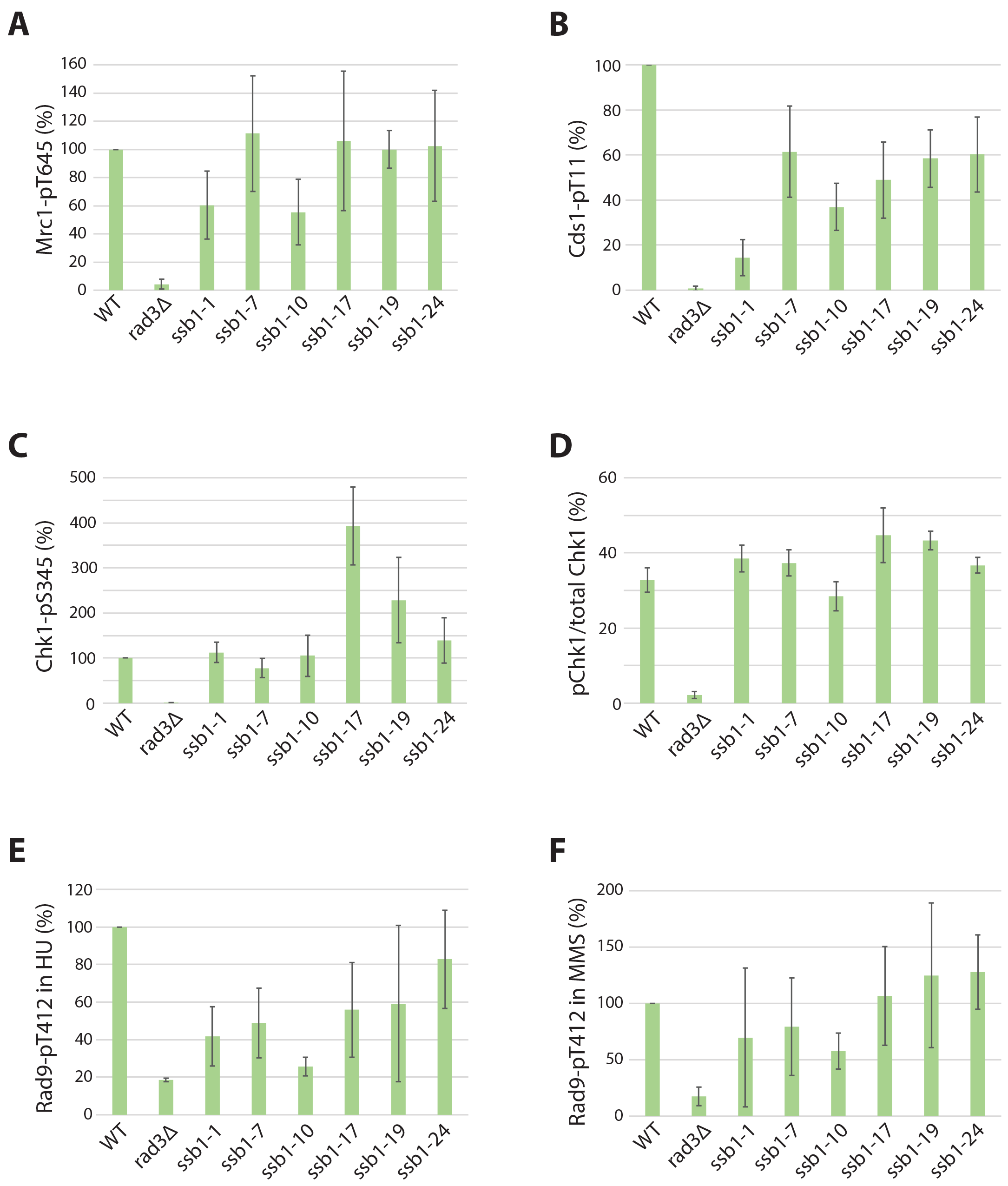

Supplement: S8 Fig — (A) Mrc1 phosphorylation in HU-treated wild type and the mutant cells was examined by Western blotting shown in Fig 6B. Quantitation results from three independent blots are shown. Error bars are means and SDs of the triplicates. (B) Quantitation results for Cds1 phosphorylation in Fig 6C from three repeats are shown. (C) Chk1 phosphorylation was examined in MMS-treated cells by Western blotting using the phospho-specific antibody as shown in Fig 6D. The quantitation results are shown from three repeats. (D) Quantitation results for Chk1 phosphorylation examined by mobility shift assay as shown in Fig 6E. (E) Rad9 phosphorylation in the 911 complex in HU-treated cells were examined as in Fig 6F. Quantitation results are from three separate blots. (F) Rad9 phosphorylation in MMS-treated cells were examined as in Fig 6G. Quantitation results are from three independent blots. (TIF) [file pgen.1010691.s008.tif]

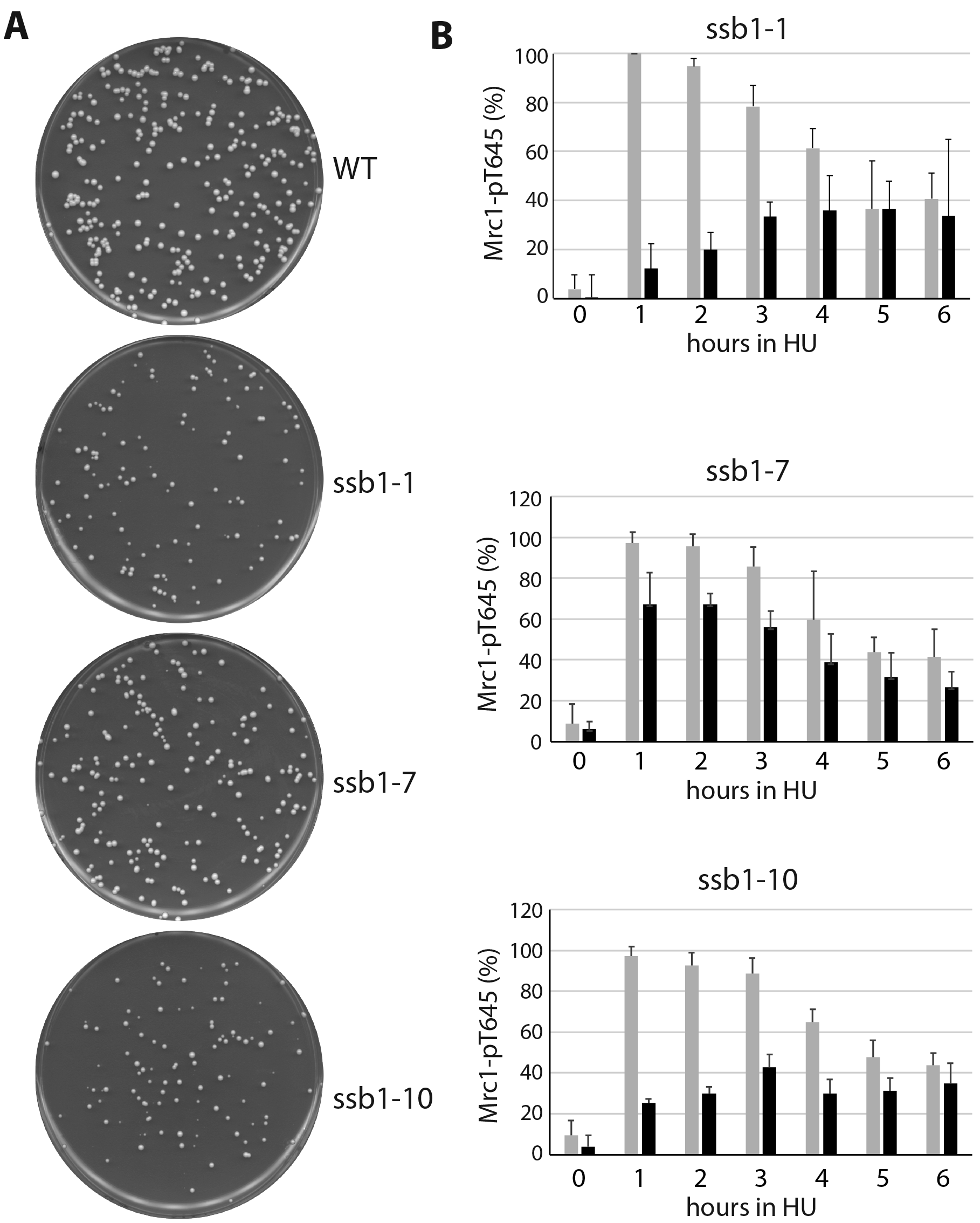

Supplement: S9 Fig — (A) Logarithmically growing wild type and the mutant cells were spread on YE6S plates. The plates were incubated at 30°C for 3 days to allow colony formation. Note: the size of ssb1-1 and ssb1-10 colonies varies significantly and is generally smaller than that of wild-type cells, showing a growth defect. (B) Time course of Mrc1 phosphorylation in HU-treated wild type (grey columns) and the mutant (black columns) cells were analysed by Western blotting as in Fig 7C. Error bars are means and SDs of triplicates. (TIF) [file pgen.1010691.s009.tif]

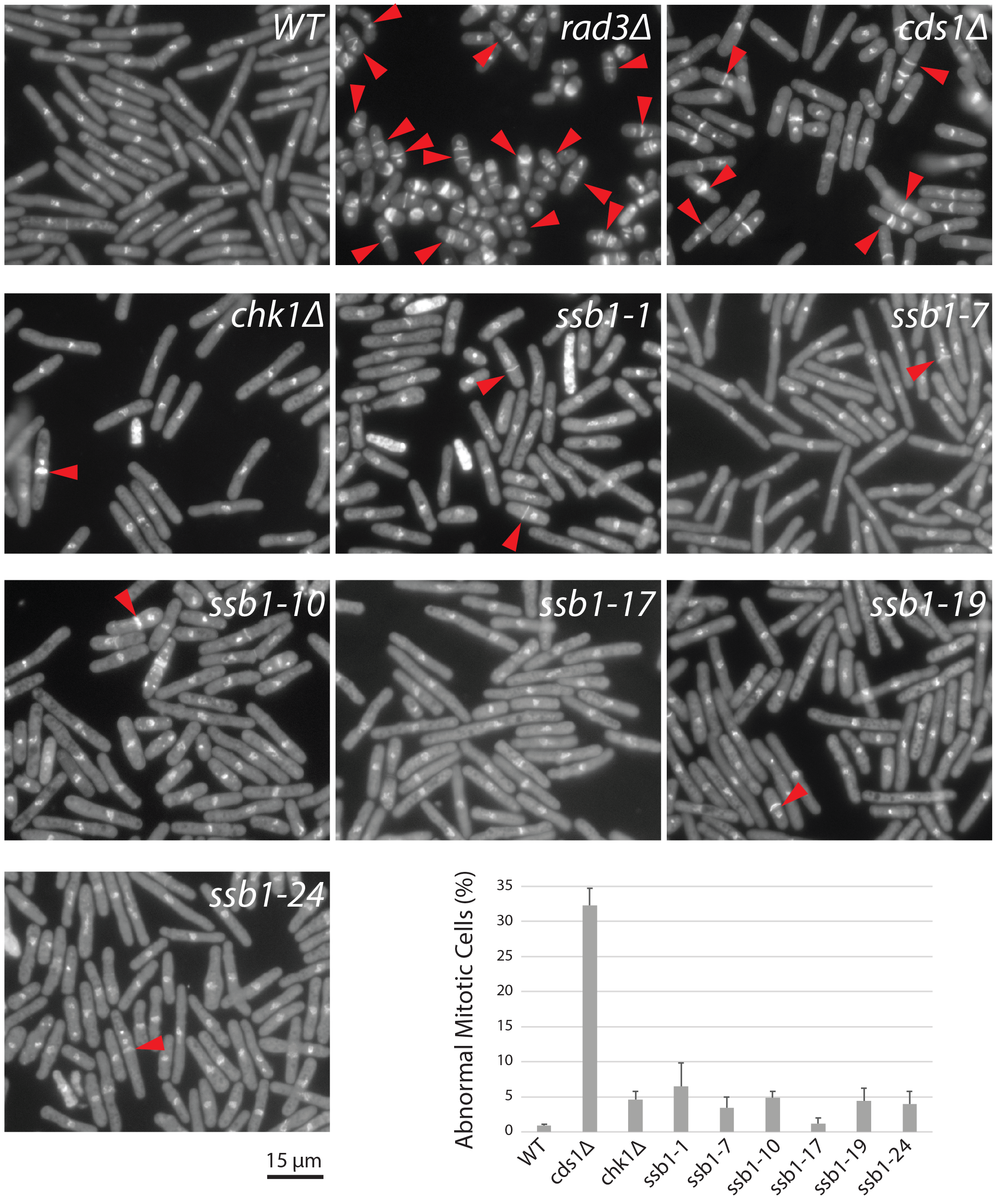

Supplement: S10 Fig — In the presence of HU, ssb1-1 and ssb1-10 mutants showed premature mitotic or cut cells in HU (red arrows) and the numbers of abnormal mitotic cells are higher than or similar to that in chk1Δ cells. Wild type, rad3Δ, cds1Δ, chk1Δ, and the six ssb1 integrant mutant cells were treated with 15 mM HU for 6 h, double-stained with Hoechst and Blankophor, and then examined under the microscope. The cut cells were counted for wild-type, cds1Δ, chk1Δ and the six ssb1 mutants in a total of ≥ 150 cells for each sample, repeated the counting three times, and presented in percentages shown in the bottom right. Error bars represent the means and SDs of triplicates. (TIF) [file pgen.1010691.s010.tif]

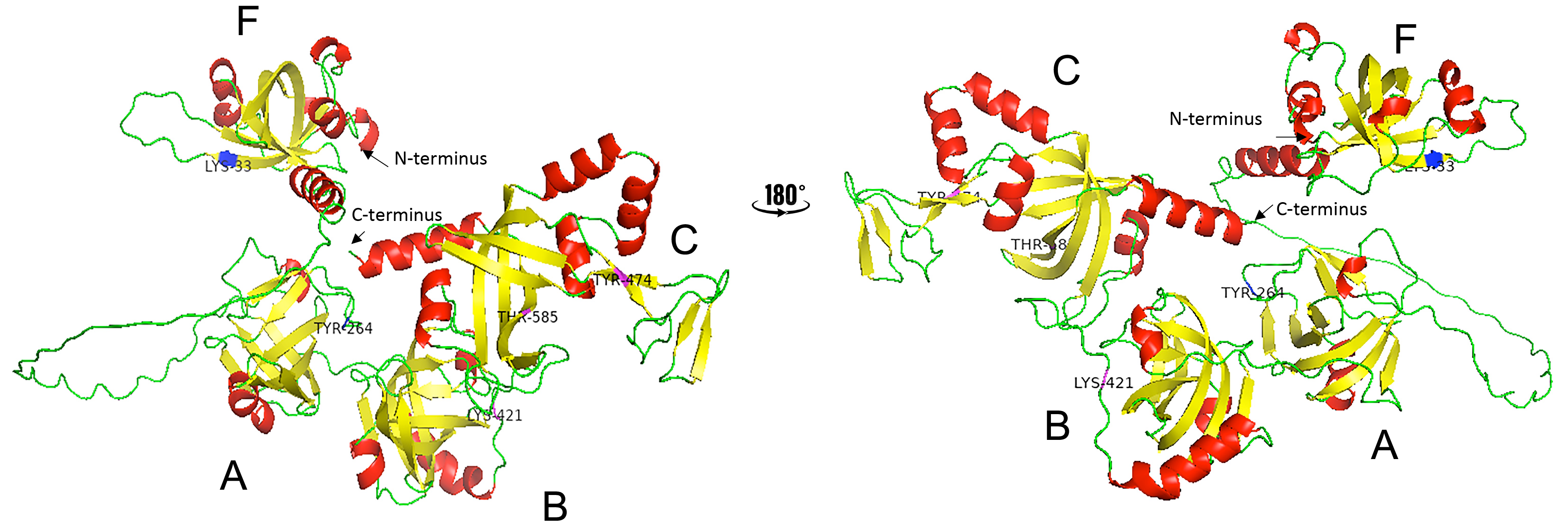

Supplement: S11 Fig — Ssb1 Alphafold secondary structure (1–609 aa) was downloaded from the Pombase (https://www.pombase.org/gene/SPBC660.13c) and edited using PyMOL software [64]. The α-helices, β-sheets, and loops are colored in red, yellow, and green, respectively, in the four conserved DNA binding domains F, A, B, and C. The N- and C-termini are indicated by arrows. The mutated residues K33 and Y264 in ssb1-1 are indicated by blue and the residues K421, Y474, and T585 in ssb1-10 are shown in magenta. (TIF) [file pgen.1010691.s011.tif]
