# Supplementary material for: Comprehensive mutational analysis of the checkpoint signaling function of Rpa1/Ssb1 in fission yeast
Source: PLoS Genet. 2023 May 18;19(5):e1010691. doi: 10.1371/journal.pgen.1010691 (PMC10231789; doi:10.1371/journal.pgen.1010691)
Supplement: S1 Table — (DOC) [file pgen.1010691.s012.doc]

**S1 Table. List of *S. pombe* strains used in this study.**

| **Strain** | **Genotype** | **Sources** |
| --- | --- | --- |
| TK7 | *h- leu1-32 ura4-D18 ade6-M210* | Lab stock |
| YJ1443 | *h- leu1-32* | Lab stock |
| TK48 | *h- leu1-32 ade6-M216* | Lab stock |
| NR1826 | *h- rad3::ura4 leu1-32 ura4-D18 ade6* | Russell lab |
| GBY191 | *h^+^ cds1::ura4 leu1-32 ura4-D18 ade6* | Kelly lab |
| TK197 | *h^+^ leu1-32 ura4-D18 ade6-M210* | Kelly lab |
| EN3239 | *h^+^ rad11A(R339H) leu1-32 ura4-D18 ade6-M210* | Russell lab |
| SC4501 | *h- ssb1-D223Y leu1-32 ura4-D18 ade6-M210* | Russell lab |
| FY20991 | *h^+^ ssb1-418(G73E) leu1-32* | YGRC |
| SC4461 | *h- ssb3::KanMX6* | Russell lab |
| NW223 | *h^+^ chk1-3HA(int) leu1-32 ade6-M216* | Walworth lab |
| NW444 | *h? chk1-S345A-3HA(int) leu1-32 ade6-M216* | Walworth lab |
| YJ15 | *h+ mrc1::ura4 leu1-32 ura4-D18 ade6-M210* | Xu lab |
| YJ1515 | *h+ tel2-C307Y-3HA:ura4 leu1-32 ade6-M210* | Xu lab |
| YJ374 | *h^+^ cds1-6his2HA(int) leu1-32 ura4-D18 ade6-M216* | Xu lab |
| YJ892 | *h^+^ 3HA-rad9(int) leu1-32 ura4-D18 ade6-704* | Xu lab |
| YJ1523 | *h^+^ ∆rad3::ura4 3HA-rad9(int) leu1-32 ura4-D18 ade6* | Xu lab |
| LLD3427 | *h- chk1-9myc2HA6his:ura4 leu1-32 ura4-D18* | Russell lab |
| YJ400 | *h? ∆rad3::ura4 cds1-6his2HA(int) leu1-32 ura4-D18 ade6-M216* | Xu lab |
| YM526 | *h^+^ ∆rad3::ura4 chk1-9myc2HA6his:ura4 leu1-32 ura4-D18* | Xu lab |
| YJ1475 | *h^+^ tel2-C307Y cds1-6his2HA(int) leu1-32 ura4-D18 ade6-M210* | Xu lab |
| YJ1754 | *h+ ssb1-R339H chk1-9myc2HA6his:ura4 leu1-32 ura4-D18* | This study |
| YJ1757 | *h+ ssb1-R339H cds1-6his2HA(int) leu1-32 ura4-D18* | This study |
| YJ1722 | *h+ ssb1-D223Y cds1-6his2HA(int) leu1-32 ura4-D18 ade6-M216* | This study |
| YJ1755 | *h- ssb1-D223Y chk1-9myc2HA6his:ura4 leu1-32 ura4-D18 ade6(?)* | This study |
| YJ1753 | *h- ssb1-G78E chk1-9myc2HA6his:ura4 leu1-32 ura4-D18 ade6(?)* | This study |
| YJ1758 | *h+ ssb1-G78E cds1-6his2HA(int) leu1-32 ura4-D18 ade6(?)* | This study |
| YJ1752 | *h- ssb3::KanMX6 chk1-9myc2HA6his:ura4 leu1-32 ura4-D18 ade6(?)* | This study |
| YJ1756 | *h- ssb3::KanMX6 cds1-6his2HA(int) leu1-32 ura4-D18 ade6(?)* | This study |
| YJ1703 | *h^+^ ssb1(R46E)-3HA:kanMX6(int) leu1-32 ura4-D18 ade6-M210* | This study |
| YJ1902 | *h^+^ ssb1-K33E-Y264H:kanMX6(int) leu1-32 ura4-D18* | This study |
| YJ1904 | *h- ssb1-R11C-C69S-D223N:kanMX6(int) leu1-32 ura4-D18* | This study |
| YJ1906 | *h- ssb1-K421R-Y474N-T585I:kanMX6(int) leu1-32 ura4-D18* | This study |
| YJ1908 | *h+ ssb1-Y57N-Q130H-N134D:kanMX6(int) leu1-32 ura4-D18* | This study |
| YJ1910 | *h+ ssb1-L35Q-P39T-N56I-G142V:kanMX6(int) leu1-32 ura4-D18* | This study |
| YJ1841 | *h+ ssb1-L100F-G119D:kanMX6(int) leu1-32 ura4-D18* | This study |
| YJ1901 | *h^+^ ssb1-K33E-Y264H:kanMX6(int) chk1-9myc2HA6his:ura4 leu1-32 ura4-D18 ade6(?)* | This study |
| YJ1911 | *h^+^ ssb1-K33E-Y264H:kanMX6(int) cds1-6his2HA(int) leu1-32 ura4-D18 ade6-M216* | This study |
| YJ1916 | *h- ssb1-K33E-Y264H:kanMX6(int) 3HA-rad9(int) leu1-32 ura4-D18 ade6* | This study |
| YJ1903 | *h- ssb1-R11C-C69S-D223N:kanMX6 chk1-9myc2HA6his:ura4 leu1-32 ura4-D18 ade6* | This study |
| YJ1912 | *h- ssb1-R11C-C69S-D223N:kanMX6 cds1-6his2HA(int) leu1-32 ura4-D18 ade6-M216* | This study |
| YJ1917 | *h- ssb1-R11C-C69S-D223N:kanMX6(int)3HA-rad9(int) leu1-32 ura4-D18 ade6* | This study |
| YJ1905 | *h+ ssb1-K421R-Y474N-T585I:kanMX6 chk1-9myc2HA6his:ura4 leu1-32 ura4-D18ade6* | This study |
| YJ1913 | *h- ssb1-K421R-Y474N-T585I:kanMX6(int) cds1-6his2HA leu1-32 ura4-D18 ade6* | This study |
| YJ1918 | *h- ssb1-K421R-Y474N-T585I:kanMX6(int) 3HA-rad9 leu1-32 ura4-D18 ade6* | This study |
| YJ1907 | *h+ ssb1-Y57N-Q130H-N134D:kanMX6 chk1-9myc2HA6his:ura4 leu1-32 ura4-D18ade6* | This study |
| YJ1914 | *h+ ssb1-Y57N-Q130H-N134D:kanMX6 cds1-6his2HA leu1-32 ura4-D18 ade6* | This study |
| YJ1919 | *h+ ssb1-Y57N-Q130H-N134D:kanMX6(int) 3HA-rad9 leu1-32 ura4-D18 ade6* | This study |
| YJ1909 | *h+ ssb1-L35Q-P39T-N56I-G142V:kanMX6 chk1-9myc2HA6his:ura4 leu1-32 ura4-D18* | This study |
| YJ1915 | *h+ ssb1-L35Q-P39T-N56I-G142V:kanMX6 cds1-6his2HA leu1-32 ura4-D18 ade6* | This study |
| YJ1920 | *h+ ssb1-L35Q-P39T-N56I-G142V:kanMX6(int) 3HA-rad9 leu1-32 ura4-D18 ade6* | This study |
| YJ1831 | *h- ssb1-L100F-G119D(int) cds1-6his2HA ura4-D18 ade6-M216* | This study |
| YJ1832 | *h+ ssb1-L100F-G119D(int) chk1-9myc2HA6his:ura4 leu1-32 ura4-D18* | This study |
| YJ1833 | *h- ssb1-L100F-G119D(int) 3HA-rad9 leu1-32 ura4-D18* | This study |

Note: The primary *ssb1* mutants #2 - #6, #8 - #9, #11 - #16, #18, #20 - #23, and #25 used in this study are not listed.
