# Supplementary material for: Comprehensive mutational analysis of the checkpoint signaling function of Rpa1/Ssb1 in fission yeast
Source: PLoS Genet. 2023 May 18;19(5):e1010691. doi: 10.1371/journal.pgen.1010691 (PMC10231789; doi:10.1371/journal.pgen.1010691)
Supplement: S2 Table — (DOCX) [file pgen.1010691.s013.docx]

**S2 Table. List of plasmids used in this study.**

| **Name** | **Description** | **Sources** |
| --- | --- | --- |
| pYJ1782 | *prom-ssb1(cDNA)/LEU2* | This study |
| pYJ1786 | *pGEM(prom-ssb1-3HA-nmtT:kanMX6-term)* | This study |
| pYJ1787 | *pGEM(prom-ssb1(K45E)3HA-nmtT:kanMX6-term)* | This study |
| pYJ1788 | *pGEM(prom-ssb1(R46E) 3HA-nmtT:kanMX6-term)* | This study |
| pYJ1789 | *pGEM(prom-ssb1(K45E-R46E) 3HA-nmtT:knaMX6-term)* | This study |
| pYJ1791 | *pBG100(6his-ssb1)* | This study |
| pYJ1632 | *prom-ssb1-term/LEU2* | This study |
| pYJ1827 | *pGEM(prom-ssb1(L100F-G119D)-3HA-nmtT:kanMX6-term)* | This study |
| pYJ1919 | *pGEM(prom-ssb1(Y57N-Q130H-N134D)-nmtT:kanMX6-term)* | This study |
| pYJ1920 | *pGEM(prom-ssb1(K33E-Y264H)-nmtT:kanMX6-term)* | This study |
| pYJ1921 | *pGEM(prom-ssb1(R11C-C69S-D223N)-nmtT:kanMX6-term)* | This study |
| pYJ1922 | *pGEM(prom-ssb1(L35Q-P39T-N56I-G142V)-nmtT:kanR-term)* | This study |
| pYJ1923 | *pGEM(prom-ssb1(K421R-Y474N-T585I)-nmtT:kanMX6-term)* | This study |
