# Supplementary material for: Comprehensive mutational analysis of the checkpoint signaling function of Rpa1/Ssb1 in fission yeast
Source: PLoS Genet. 2023 May 18;19(5):e1010691. doi: 10.1371/journal.pgen.1010691 (PMC10231789; doi:10.1371/journal.pgen.1010691)
Supplement: S3 Table — (DOCX) [file pgen.1010691.s014.docx]

**S3 Table. List of PCR and sequencing primers used in this study.**

| **Name** | **Sequence** (5´ -> 3´) | **Note** | |
| --- | --- | --- | --- |
| 5’Rpa1(NdeI)f | GATAggATCCGTTTGTTTTGCTTCC | | Cloning & Colony PCR |
| 3’Rpa1(XmaI)r | aacaccccgggTTATTGAGCAGACTCAATGAAA | | Cloning |
| Rpa1(P)SphI | ttacgcatgcgagctaaaagagaggt | | Cloning |
| SpRpa1(b)SacI-b | gtctttGAgctcagttatattattatt | | Cloning |
| Rpa1(P)f | GGCGAACTGTAGTGGATAATCTG | | Sequencing & Colony PCR |
| Rpa1(494-513)f | TGATGAAGAAGCCAGCAGCG | | Sequencing |
| Rpa1(1001-18)f | GTGGATTTGACAAGCGTG | | Sequencing |
| Rpa1(594-576)r | AACACGGGCACGAATAGTC | | Sequencing & Colony PCR |
| Rpa1(1156-38)r | AAGAGCGTCCTTGAAAGTC | | Sequencing |
| Rpa1(1506-23)f | TGATGCTCCTCAATACCG | | Sequencing |
| Rpa1(T)r | ttactcaattaaagaatgtaacc | | Sequencing & Colony PCR |
| Rpa1(NotI)-b | acacgcggccgccTTGAGCAGACTCAATGAAAT | | C-term tagging |
| Ura4-b2 | CTACCAATTCTAAGATTTCGGATTTC | | Colony PCR |
| 3´ura4 | GCAATTTCTATGCGCACCCGTTCTCGGAGC | | Colony PCR |
| nmtTERM-r | GGGCTTCCATAGTTTGAAAG | | Colony PCR |
