# Supplementary material for: Comprehensive mutational analysis of the checkpoint signaling function of Rpa1/Ssb1 in fission yeast
Source: PLoS Genet. 2023 May 18;19(5):e1010691. doi: 10.1371/journal.pgen.1010691 (PMC10231789; doi:10.1371/journal.pgen.1010691)
Supplement: Attachment — Submitted filename: Response to Reviewers comments.pdf [file pgen.1010691.s016.pdf]

2023-PGenetics “**Comprehensive mutational analysis of the checkpoint signaling function of Rpa1/Ssb1 in fission yeast**”

Response to reviewers’ comments

Note: Reviewers’ comments are colored **blue** and the response **black**.

Reviewer #1: RPA is a conserved protein complex that specifically binds to ssDNA. Because ssDNA represents an important intermediate in DNA replication, recombination, and damage repair, RPA plays a crucial role in DNA metabolism. At the stalled replication forks, ssDNA-associated RPA recruits not only DNA polymerases to facilitate DNA replication but also checkpoint proteins for checkpoint activation and cell cycle arrest. However, separation-of-function mutants of RPA in DNA metabolism and checkpoint control are unavailable. RPA consists of three subunits, Rpa1, Rpa2, and Rpa3, and all are essential. This manuscript described a genetic screen for rpa1 (ssb1) mutants in fission yeast that show sensitivity to DNA synthesis inhibitor HU and DNA damage agent MMS. Among the isolated 25 ssb1 mutants, two of them exhibited partial checkpoint defect in response to DNA replication stress. These mutants will be important for the functional studies of RPA, but some issues need to be addressed before publication.

Major points:

Some of the conclusions are not essay to follow. For example, based on the evidence, it is difficult to understand the statement that “the HU sensitivity observed in the four RPA mutants in Fig. 1A is likely a combinatory effect of the minimal replication defects and the HU-induced oxidative stress” in page 10. More rationalization is needed for this statement.

Response: We appreciate the reviewer positive comments and suggestions and have rewritten this chronic cell-killing effect to the four previously reported RPA mutants in fission yeast (see highlighted lines 223-226 on p10 in the revised manuscript).

Presented evidence in this manuscript indicates that the second mutation outside ssb1 contributes to MRC defect in ssb1-24 mutant. Therefore, the description about this mutant needs to be shortened.

Response: We agree with the reviewer and this section has been shortened as suggested (see highlighted lines 365-378 on p17).

Fig. 3C and D show different results about Chk1 phosphorylation (band-shift vs phospho-antibody) in ssb mutants. For example, Chk1 phosphorylation at S345 was decreased in MMS-treated ssb1-10 cells using anti pS345 antibody, but the phosphorylation-induced Chk1 band-shift in ssb1-10 cells remained relatively consistent. One possibility is that the band-shift is a result of multiple phosphorylation events by multiple kinases, thus it is not sensitive enough to detect the specific phosphorylation at S345. It is unclear if this difference was observed in other labs. More discussion for this difference is needed, or it is hard to conclude whether DDC activity is affected by ssb1 mutations.

Response: We have been using the mobility shift assay for Chk1 activation in the past and the initial stage of the screen in this study. Because of the negative results, we were concerned with the sensitivity of this assay and therefore, generated the phosphor-specific antibody. To our knowledge, phosphor-specific antibodies for fission yeast Chk1-pS345 have been reported only by the laboratories of Nancy Walworth and Paul Russell over 20 years ago. However, for unknown reasons, they stopped using the phosphor-specific antibodies after their initial studies and started using the mobility shift assay in their following up studies in the past. We have tried the phosphor-specific antibody by the Walworth lab

before. The specificity is however not very impressive, probably due to the long-term storage in the freezer. In contrast, our newly made phosphor-specific antibody against Chk1-pS345 appears to be too sensitive that generates significant variations between experiment repeats as we discussed in the Discussion. One possible reason is that unlike other phosphorylated proteins, unphosphorylated Chk1 and the phosphorylated Chk1 move in a very sharp bands in SDS-PAGE gel, which makes it more challenging to accurately quantitate the band intensities. Of course, the loading can be another issue for IPed samples. We also noticed the low level of Chk1-pS345 signal in the #10 primary mutant and believe that it is likely the technical issue during the initial stage of the screen because: (1) the repeated experiments with mutant integrants in Figure 6D and S8C show slightly lower or wt levels of phosphorylation; (2) the mobility shift assay has been proven sensitive enough to detect Chk1 phosphorylation by multiple labs in the past, including my own lab. The advantage of using this assay is that the loading is never a concern. Using this method, the results in Fig,4D and 6E are consistent, ie, no significant reduction in the #10 mutant. (3) The #10 mutant elongates in HU, which is consistent with Chk1 activation by fork damage. Nevertheless, following the reviewer's suggestion, we added a comment on the difference between Fig. 4C and 6D in the revised manuscript (see highlighted lines 392-395 on p18).

One interesting observation is that ssb1-1 mutant is sensitive to both DNA replication stress (HU) and DNA damage (MMS, CPT, Bleomycin). Although the phosphorylation of Cds1 and Mrc1 was decreased in ssb1-1 cells in response to DNA replication stress, the number of ssb1-1 cells with cut phenotype was low (~5%). The authors speculated that activation of DDC (Chk1) likely contributes to the low number of cells with cut phenotype. This speculation can be tested by analyzing the cut phenotype in ssb1-1 chk1Δ cells treated with HU and DNA damaging agents. The analysis of the phenotypes of this double mutant could be informative.

Response: We agree with the reviewer. The results should be expected and might be informative. We will include this idea in our future experiments.

In Fig. 5, it would be better to show quantitative analysis of the phosphorylation of Mrc1, Cds1, and Chk1 in ssb1-1/10 mutants.

Response: The quantitative results the phosphorylation of Mrc1, Cds1, and Chk1 in ssb1-1/10 mutants are shown in Figure S8A-D.

Minor points

It seems that there is a dashed line on the top of Fig. 1L.

Fixed.

The conclusion from Fig. 6D (FACS data) was not clearly described.

We rewrote this part, see lines 424-433 on p19.

More explanation of the difference between DRC and DDC is needed in the introduction section as this is critical to understand the function of RPA in checkpoint signaling.

A little more details about DRC and DDC are added (see lines 85-87 on p4).

Line 108: it is better to use DRC but not S phase checkpoint pathway.

Fixed.

Line 133: to screen mutants in the large RPA subunit ssb1

Fixed.

Line 185: “their mutations” should be “these mutants”

Fixed.

Line 218: a brief description of the method in Fig. 1J is necessary here.

Yes, added (see line 218-219 on p10).

Line 240: The statement “consistent with its major role in cells lacking ssb3” is difficult to follow.

This confusing statement is deleted (see line 242-243 on p11).

Line 207: “to screening” should be “to screen”

Fixed.

Line 223: oxidative

Fixed.

Line 275: random

Fixed.

Line 331: Change to “Mrc1 and Cds1, particularly Cds1, was reduced…”

Fixed.

Line 367: changed to “HU-sensitive (hus) phenotype

Added.

Line 423: FACS needs to be mentioned here for the cell cycle progression analysis

Added.

Line 438: change to “the DDC remains activated…”

Fixed.

Line 440: specify the number of cells with cut phenotype here.

Added.

Line 464: a genetic screen

Fixed.

Line 473: screen

Fixed.

Supplemental

Fig. S1: phospho-specific

Fixed.

Reviewer #2: This paper presents a comprehensive mutational analysis of the large subunit of RPA in fission yeast, in the effort to identify mutants that specifically disrupt the replication checkpoint. This is based on the ability of RPA to signal via ATRIP to the ATR kinase. This work is nicely performed. Only a couple of alleles with minor checkpoint defects were identified. Presumably, none of these point mutations is sufficient to disrupt ATRIP signally; presumably this could be observed with a Rad26-GFP fusion.

The authors should cite, and discuss, work from cerevisiae describing RPA as itself a checkpoint substrate, meaning it may function downstream as well as upstream, potentially confounding the analysis. Aside from these minor concerns the work is adequately done.

Response: We really appreciate the advice as we should have cited those works before we submitted the manuscript. In this revised manuscript, three early studies on the phosphorylation of Rfa1 and Rfa2 by Mec1 in budding yeast are cited (see lines 95, 98-100, and 111-112 on p5 and references 21, 22, and 25).

Reviewer #3: Xu et al described the identification of fission yeast Rpa1 mutations that impair DNA replication checkpoint (DRC) but not the DNA damage checkpoint (DDC). RPA is involved many DNA transactions, such as DNA replication, DNA repair pathways, and checkpoint responses. Commonly used RPA mutant alleles in budding and fission yeasts tend to have defects in many processes and manifest highly complex phenotype. When RPA mutants impair DNA replication and repair, cells sustain more lesions, which can lead to stronger DDC and DRC responses. Consequently, despite the widely held belief that RPA is needed for DDC/DRC, available RPA mutants do not reduce DDC and DRC levels. The authors confirmed proficient DDC/DRC in ssb1-R229H, D223Y, and G79E mutants (all have ≥ 2X protein level reduction) in HU and MMS, and that they showed chronic but not acute sensitivity toward HU.

Addressing the contradiction requires separation-of-function RPA alleles that specifically impair DDC and/DRC. The authors first tried to generate rfa1-t11 (K45E) equivalent mutations, but three types of mutations all caused lethality or severe growth defect. Thus, they performed PCR-based mutagenesis of Ssb1 and integrated the mutated alleles into chromosome using pop-in and pop-out method. Among 25 mutants, the authors found six showed some defects suggestive for DRC impairment, further analyses using integrated alleles found that ssb1-1 (K33E, Y264H) and -10 (K421R-Y474N-T585I) met the DRC impairment criteria better than to others, and also showed stronger bleomycin sensitivity than rad3Δ and exhibit slower growth, suggesting for replication and DSB repair defects.

While the data suggest that the ssb alleles were not be the ideal separation of function alleles, demonstrating they indeed reduce DRC helps to address the conundrum described above regarding the role of RPA in checkpoint. The study was done thoroughly and both the results and mutants generated can be useful to the field. I have two main suggestions.

1) Given the large numbers of RPA mutants examined, it is useful to map some of them, particularly, ssb1-1 and 10, on a 3D structure of RPA, which can be generated by the alpha-fold program. Are the mutations located in the area involved in binding to ssDNA and/or ATRIP/Rad26/Ddc2? This excise may

help to provide plausible explanations regarding why specific alleles impair DRC while many others do not.

Response: We appreciate the suggestion and added a new figure in Figure S11 in the revised manuscript.

2) ssb1-1 and -10 likely affect DNA replication and break repair; authors might want to try to separate the mutations within each allele and see if a true separation of function allele with specific DRC defect can be obtained. The authors seemed to have already generated such alleles, it would be informative to include those data/

Response: We agree with the reviewer. This will be included in our future experiments.

Minor point. - Figure 1 needs to be made into two separate figures.

Response: Following the advice, we have separated Figure 1 into Figure 1 and 2. Therefore, there are seven main figures in the revised manuscript.
