# Supplementary material for: Comprehensive mutational analysis of the checkpoint signaling function of Rpa1/Ssb1 in fission yeast
Source: PLoS Genet. 2023 May 18;19(5):e1010691. doi: 10.1371/journal.pgen.1010691 (PMC10231789; doi:10.1371/journal.pgen.1010691)
Supplement: Attachment — Submitted filename: 2023-PGENETICS-D-23-00250.docx [file pgen.1010691.s015.docx]

2023-PGenetics

RPA is a conserved protein complex that specifically binds to ssDNA. Because ssDNA represents an important intermediate in DNA replication, recombination, and damage repair, RPA plays a crucial role in DNA metabolism. At the stalled replication forks, ssDNA-associated RPA recruits not only DNA polymerases to facilitate DNA replication but also checkpoint proteins for checkpoint activation and cell cycle arrest. However, separation-of-function mutants of RPA in DNA metabolism and checkpoint control are unavailable. RPA consists of three subunits, Rpa1, Rpa2, and Rpa3, and all are essential. This manuscript described a genetic screen for rpa1 (ssb1) mutants in fission yeast that show sensitivity to DNA synthesis inhibitor HU and DNA damage agent MMS. Among the isolated 25 ssb1 mutants, two of them exhibited partial checkpoint defect in response to DNA replication stress. These mutants will be important for the functional studies of RPA, but some issues need to be addressed before publication.

Major points:

Some of the conclusions are not essay to follow. For example, based on the evidence, it is difficult to understand the statement that “the HU sensitivity observed in the four RPA mutants in Fig. 1A is likely a combinatory effect of the minimal replication defects and the HU-induced oxidative stress” in page 10. More rationalization is needed for this statement.

Presented evidence in this manuscript indicates that the second mutation outside ssb1 contributes to MRC defect in ssb1-24 mutant. Therefore, the description about this mutant needs to be shortened.

Fig. 3C and D show different results about Chk1 phosphorylation (band-shift vs phospho-antibody) in ssb mutants. For example, Chk1 phosphorylation at S345 was decreased in MMS-treated ssb1-10 cells using anti pS345 antibody, but the phosphorylation-induced Chk1 band-shift in ssb1-10 cells remained relatively consistent. One possibility is that the band-shift is a result of multiple phosphorylation events by multiple kinases, thus it is not sensitive enough to detect the specific phosphorylation at S345. It is unclear if this difference was observed in other labs. More discussion for this difference is needed, or it is hard to conclude whether DDC activity is affected by ssb1 mutations.

One interesting observation is that ssb1-1 mutant is sensitive to both DNA replication stress (HU) and DNA damage (MMS, CPT, Bleomycin). Although the phosphorylation of Cds1 and Mrc1 was decreased in ssb1-1 cells in response to DNA replication stress, the number of ssb1-1 cells with cut phenotype was low (~5%). The authors speculated that activation of DDC (Chk1) likely contributes to the low number of cells with cut phenotype. This speculation can be tested by analyzing the cut phenotype in ssb1-1 chk1Δ cells treated with HU and DNA damaging agents. The analysis of the phenotypes of this double mutant could be informative.

In Fig. 5, it would be better to show quantitative analysis of the phosphorylation of Mrc1, Cds1, and Chk1 in ssb1-1/10 mutants.

Minor points

It seems that there is a dashed line on the top of Fig. 1L.

The conclusion from Fig. 6D (FACS data) was not clearly described.

More explanation of the difference between DRC and DDC is needed in the introduction section as this is critical to understand the function of RPA in checkpoint signaling.

Line 108: it is better to use DRC but not S phase checkpoint pathway.

Line 133: to screen mutants in the large RPA subunit ssb1

Line 185: “their mutations” should be “these mutants”

Line 218: a brief description of the method in Fig. 1J is necessary here.

Line 240: The statement “consistent with its major role in cells lacking ssb3” is difficult to follow.

Line 207: “to screening” should be “to screen”

Line 223: oxidative

Line 275: random

Line 331: Change to “Mrc1 and Cds1, particularly Cds1, was reduced…”

Line 367: changed to “HU-sensitive (hus) phenotype

Line 423: FACS needs to be mentioned here for the cell cycle progression analysis

Line 438: change to “the DDC remains activated…”

Line 440: specify the number of cells with cut phenotype here.

Line 464: a genetic screen

Line 473: screen

Supplemental

Fig. S1: phospho-specific
